# Complete chloroplast genome characterization and phylogenetic analysis of two natural caffeine-free *Camellia yungkiangensis* H. T. Chang accessions

**DOI:** 10.3389/fpls.2026.1807875

**Published:** 2026-04-28

**Authors:** Sihui Liang, Chun Yang, Xiaozeng Mi, Mengsha Tang, Yan Li, Anran Wang, Can Guo, Zhengwu Chen, Dahe Qiao

**Affiliations:** 1 Guizhou Tea Research Institute, Guizhou Academy of Agricultural Sciences, Guiyang, Guizhou, China; 2 Guizhou Key Laboratory of Molecular Breeding for Characteristic Horticultural Crops, Guiyang, Guizhou, China; 3 Ministry of Agriculture and Rural Affairs Key Laboratory of Crop Genetic Resources and Germplasm Innovation in Karst Region, Guiyang, Guizhou, China

**Keywords:** caffeine-free, *Camellia yungkiangensis*, chloroplast genome, phylogenetic, tea plant

## Abstract

The discovery and utilization of naturally caffeine-free tea germplasms hold significant potential for developing novel tea products tailored to health-conscious consumers. This study focuses on *Camellia yungkiangensis*, a rare caffeine-free tea plant resource, to systematically evaluate its biochemical traits, chloroplast genome architecture, and phylogenetic relationships. A total of 162 germplasm samples from five distinct populations in Guizhou, China, were analyzed for catechins and alkaloids. Results confirmed the universal absence of caffeine across all accessions, accompanied by high theobromine content (average 4%) and a unique catechins profile characterized by low epigallocatechin gallate (EGCG, <1%) and high catechin (C, >6%) levels. Two morphologically distinct types accessions—shrub (RJ) and tree (SD)—were selected for complete chloroplast genome sequencing utilizing the Oxford Nanopore sequencing platform. Both genomes exhibited a typical quadripartite structure, with total lengths of 156,872 bp (RJ) and 156,892 bp (SD), and encoded 133 identical genes. Repetitive sequence analysis identified 69 (RJ) and 70 (SD) SSRs, predominantly mononucleotide and dinucleotide repeats, and a unique hexameric SSR (AAAGAA)_3_ present exclusively in RJ serves as a distinctive molecular marker. Phylogenetic reconstruction using 55 *Camellia* species placed RJ and SD in a distinct clade closely related to *C. pitardii* and *C. danzaiensis*, highlighting their evolutionary divergence from cultivated tea plant varieties. These findings provide genomic and biochemical foundations for leveraging *C. yungkiangensis* in breeding programs aimed at expanding the diversity of specialty teas, particularly caffeine-free varieties with unique health benefits.

## 1 Introduction

Rongjiang tea (*C. yungkiangensis* H. T. Chang) is a tea plant resource named after its type specimen locality, Rongjiang county in Guizhou Province, within the taxonomic system of *Camellia* sect. *Thea* (Chang, 1981a). It was formally reported in 1981 and classified under the ser. *Gymnogynae* alongside *C. costata* (Chang, 1981b). However, subsequently, in Ming’s classification system, Rongjiang tea is classified under *C. costata* (Ming, 1992). In recent years, it has regained attention due to its naturally caffeine-free characteristics.

Tea represents one of the most widely consumed non-alcoholic beverages globally. Its distinctive flavor and health-promoting properties are largely attributed to a rich array of secondary metabolites, including tea polyphenols, theanine, and alkaloids (Mozumder et al., 2025). Caffeine is the most abundant alkaloid in tea leaves (Zhang et al., 2022). However, due to its potential adverse effects in specific populations, demand is increasing for decaffeinated tea beverages (Ashihara and Crozier, 2001). Consequently, producing naturally decaffeinated tea while retaining its original sensory characteristics and beneficial compounds presents a significant challenge (Zhang et al., 2020b; Chen et al., 2025). Although the caffeine synthase gene (*TCS1*) in tea plants has been cloned and functionally characterized (Kato et al., 2000), the absence of a stable genetic transformation system for *C. sinensis* currently impedes the development of caffeine-free tea varieties via genetic engineering. The identification of naturally occurring low-caffeine or caffeine-free tea plant resources offers a viable approach to addressing this challenges.

Given that both primary cultivated tea varieties, the *C. sinensis* var. *sinensis* (CSS) type and the *C. sinensis* var. *assamica* (CSA) type, contain substantial caffeine content, the exploration of caffeine-free tea plants from wild sources has become imperative. The initial documented natural caffeine-free resource within tea plants was *C. ptilophylla*, identified within Nankun Mountain region of Guangdong Province. Characterized by the absence of caffeine yet richnessin theobromine, this species is commonly designated as “Cocoa Tea” (Chang et al., 1988). Subsequently, numerous studies have reported the identification of low- or caffeine-free tea plant germplasms across various *Camellia* species (Tang et al., 2010; Zhang et al., 2013; Jin et al., 2014, Jin et al., 2018; Zhang et al., 2020b). Rongjiang tea represents a recently reported natural caffeine-free tea plant resource (Mi et al., 2023). However, the uniqueness of its habitat, combined with challenges such as difficulties in rooting during *ex situ* cutting propagation, natural aging and degeneration, and predominantly sterile pollen, necessitates urgent rescue and conservation measures for this resource.

Ascertaining the origin and evolutionary history of a species constitutes a fundamental prerequisite for its targeted conservation and utilization. In recent years, chloroplast genome studies have yielded considerable insights into plant phylogenetic relationships and evolutionary trajectories (Huang et al., 2014; Li et al., 2021; Zhao et al., 2022). In angiosperms, chloroplast genome sequences exhibit high levels of structural conservation, typically featuring a quadripartite circular configuration comprising a large single-copy region (LSC), a small single-copy region (SSC), and two inverted repeat regions (IRs) (Shinozaki et al., 1986). Owing to their sluggish molecular evolution and low molecular weight, these genomes offer distinct advantages for studies on cytoplasmic inheritance, plant phylogeny, DNA barcode development, genetic diversity assessment, and phylogenetic analysis (Daniell et al., 2016). This is particularly important for plants in the *Camellia* sect. *Thea*, which have numerous closely related species and are generally capable of fertile hybridization. Within *Camellia* sect. *Thea*, over 100 chloroplast genomes have been sequenced. Comparative analyses indicate that the length of the chloroplast genome in tea plants range from 153,044 and 158,916 bps, with wild tea accessions generally possessing shorter genomes than cultivated varieties (Qiao, 2024).

In this study, the sampling scope and sample size were initially expanded to determine whether the caffeine-free trait represents a common characteristic of *C. yungkiangensis* or is specific to certain accessions. Subsequently, the chloroplast genomes of both shrub- and tree- form *C. yungkiangensis* were sequenced. Comparative analysis of chloroplast genome sequences was conducted, alongside phylogenetic analysis involving other sections of the genus *Camellia*. These findings not only contribute to a more comprehensive understanding of the caffeine-free trait in *C. yungkiangensis* but also establish a foundation for evolutionary studies within the genus *Camellia* and provide support for the conservation and utilization of *C. yungkiangensis* resources.

## 2 Materials and methods

### 2.1 Plant materials

In this study, 162 germplasm resources of *C. yungkiangensis* were collected from five distinct locations: Dujiang Town (G0, 108.1624° E, 25.9920° N) in Sandu Shui Autonomous County; Jihua Township (G1, 108.3556° E, 25.7514° N), Liangwang Township (G2, 108.4779° E, 26.3427° N), and Langdong Town (G3, 108.5266° E, 26.3583° N) in Rongjiang County; and the Guizhou Tea Germplasm Resource Nursery (G, 106.6692° E, 26.4992° N) in Huaxi District, Guiyang City (**Supplementary Figure 1**). The sample sizes per location were as follows: G0 (n=53), G1 (n=48), G2 (n=18), G3 (n=35), and G (n=8). Young leaves were sampled from each accession in April 2024. As this collection was conducted exclusively for research purposes, no special permits were required. Samples were immediately flash-frozen in liquid nitrogen and subsequently stored in a -80 °C freezer for future use.

### 2.2 Extraction and detection of catechins and alkaloids

The catechins, including (−)-catechin (C), (−)-epicatechin (EC), (−)-epicatechin gallate (ECG), (−)-gallocatechin (GC), (−)-epigallocatechin (EGC) and (−)-epigallocatechin gallate (EGCG), caffeine and theobromine were analyzed using an Agilent 1260 Infinity II HPLC system (USA) equipped with a hypersil ods2 C18 column (4.6 × 250 mm, 5 μm). Extraction and analytical procedures followed our previously described methods (Yang et al., 2024). Briefly, they were extracted with 70% methanol and separated under gradient elution using ultrapure water (mobile phase A) and a mixture of N,N-dimethylformamide, methanol, and glacial acetic acid (40:2:1.5, v/v/v; mobile phase B). The flow rate was 1 mL/min, detection wavelength was set at 278 nm, the column temperature was maintained at 35 °C, and the injection volume was 5 μL. The compounds were identified by comparing retention times and UV spectra with reference standards.

### 2.3 DNA extraction and sequencing

Genomic DNA was extracted from two samples (designated SD and RJ) using the CTAB method, followed by purification with a gDNA purification kit (NEB, T3010). DNA quality was assessed via 1.0% agarose gel electrophoresis and quantified using an ND-1000 spectrophotometer (Thermo Fisher, USA). Library construction and sequencing were performed on an Oxford Nanopore PromethION platform at Wuhan Benagen Technology Co., Ltd (Wuhan, China).

### 2.4 Chloroplast genome assembly and annotation

High-quality sequencing data of 12,148 Mb and 10,967 Mb were obtained for the RJ and SD samples, respectively. *De novo* assembly was performed using Flye software (Kolmogorov et al., 2019) employing default parameters, generating graph-based assemblies in GFA format. Contig fragments harboring the chloroplast genome were identified by BLASTn (Chen et al., 2015) against published tea plant chloroplast genomes, using the parameters “-evalue 1e-5 -outfmt 6 -max_hsps 10 -word_size 7 -task blastn-short”. Visualization of the GFA file was conducted using Bandage software (Wick et al., 2015), enabling the selection of chloroplast contigs according to the BLASTn results. Chloroplast genome annotation was executed using the CPGAVAS2 software (Shi et al., 2019). tRNA genes were annotated with tRNAscan-SE (Chan et al., 2021), while rRNA genes were identified via BLASTn. Annotation errors within the chloroplast genome were manually corrected using CPGView (Liu et al., 2023) and Apollo software (Lewis et al., 2002). The circular structure of the chloroplast genome was visualized using OGDRAW with default settings (Greiner et al., 2019). The complete chloroplast genome sequences and annotations of SD and RJ have been deposited in the NCBI GenBank under the accession numbers PV424009 and PV424010, respectively.

### 2.5 Analysis of relative codon usage and repetitive sequence

For codon usage bias analysis, protein-coding sequences were extracted from the genome using Phylosuite software (Zhang et al., 2020a). Subsequently, codon usage analysis was conducted on the protein-coding genes within the chloroplast genome utilizing MEGA 7.0 software (Kumar et al., 2016), and relative synonymous codon usage (RSCU) values were calculated. For repetitive sequence analysis, simple sequence repeats (SSRs) were identified using MISA (Beier et al., 2017), while tandem repeat and dispersed repeat were analyzed using TRF (Benson, 1999) and REPuter (Kurtz et al., 2001), respectively.

### 2.6 Phylogenetic analysis

For phylogenetic analysis, complete chloroplast genomes of 55 species were retrieved from the NCBI database. This dataset comprised 53 species of *Camellia* and two *Polyspora* species designated as outgroups. Alignment of all 57 complete chloroplast genomes was performed using MAFFT software (Katoh and Standley, 2013). Phylogenetic analysis was conducted using both maximum likelihood (ML) and Bayesian inference (BI) approaches. ML analysis was executed in IQ-TREE3 (Wong et al., 2025) under the optimal TVM+F+I+R4 model, with 1000 bootstrap replicates and default setting for remaining parameters. BI analysis was implemented in MrBayes v3.2.7a (Ronquist et al., 2012) using the GTR+I+G model, running 2,000,000 generations, and discarding the initial 25% of samples as burn-in. Resultant phylogenetic trees were visualized employing the iTOL software platform (Letunic and Bork, 2019).

## 3 Results and discussion

### 3.1 Caffeine-free trait is universal in *C. yungkiangensis*

**Figure 1:**
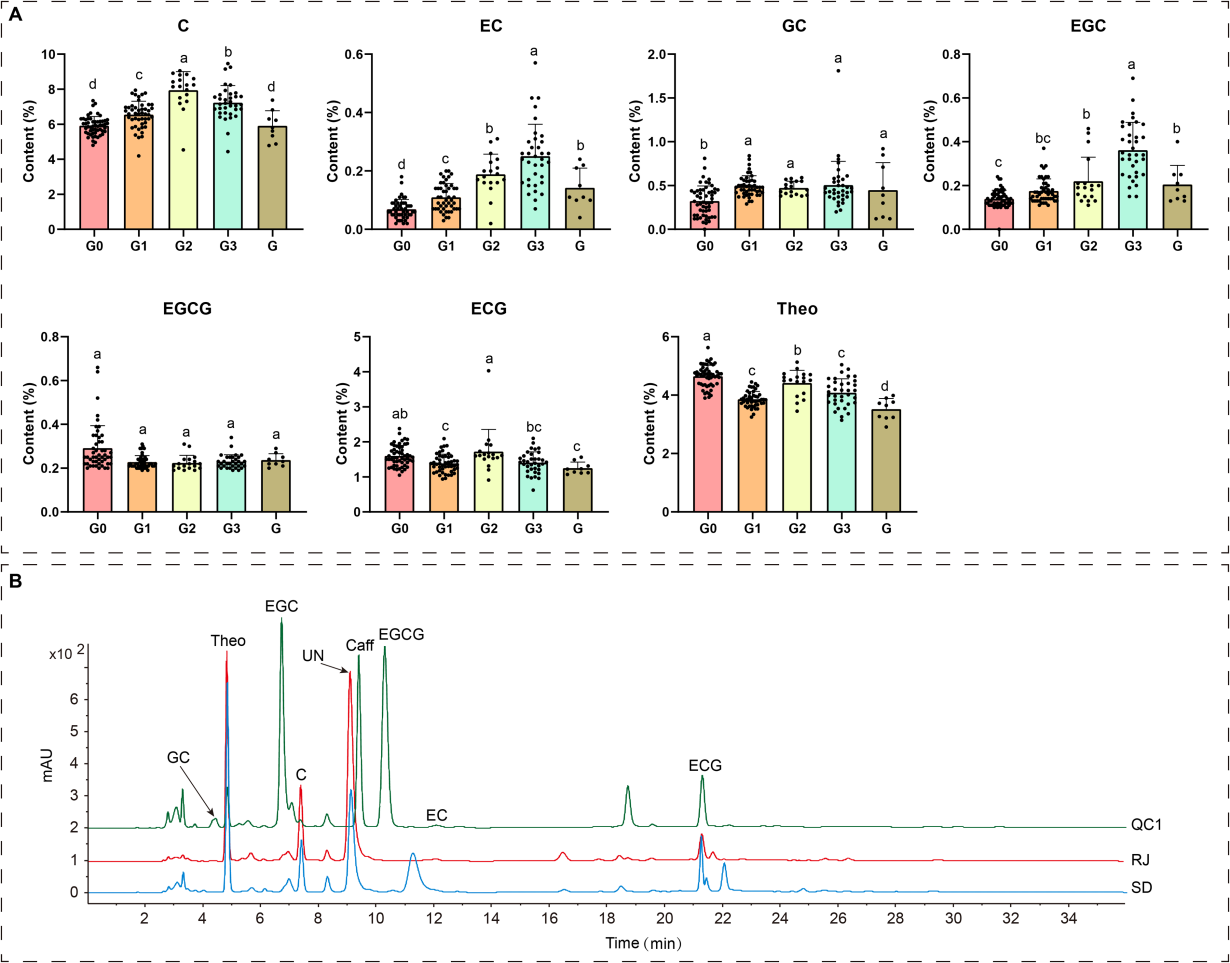
Catechins and theobromine content in different *C. yungkiangensis* accessions. **(A)** Catechins and theobromine levels across distinct *C. yungkiangensis* populations. **(B)** Representative liquid chromatography chromatograms of catechins, theobromine, and caffeine in young leaves from two *C. yungkiangensis* accessions (RJ and SD) and the cultivated tea plant variety ‘Qiancha 1’ (QC1). C, catechin; EC, epicatechin; GC, gallocatechin; EGC, epigallocatechin; EGCG, epigallocatechin gallate; ECG, epicatechin gallate; Theo, theobromine; Caff, caffeine; mAU, milli-absorbance unit; UN, unknown.

In previous study, we identified a naturally caffeine-free tea plant resource (*C. yungkiangensis*) (Mi et al., 2023). To ascertain whether this caffeine-free trait represents an isolated occurrence within this specific resource or a common characteristic across such resources, we expanded our collection scope. This led to the identification of four distinct populations exhibiting significant geographical isolation (designated G0-G3). In addition, we sampled eight accessions (designated G) that had been propagated via cuttings and transplanted to a different location earlier. A total of 162 samples were detected for catechin components, caffeine, theobromine, and theacrine content. The results showed that none of these accessions contained detectable levels of caffeine or theacrine. Instead, they were characterized by high theobromine content. Theobromine levels in the 162 samples ranged from 2.90% to 5.57%, averaging 4.23% of dry weight (**Figure 1**). This demonstrates that the caffeine-free trait is a shared characteristic among this category of resources and is not compromised by variations in growing environments. Notably, besides being caffeine-free, these accessions contained extremely low levels of EGCG (<1%) but high levels of C (>6%). This compositional profile contrasts markedly with cultivated tea plants, which are typically rich in EGCG but contain minimal C (Fu et al., 2024).

### 3.2 Chloroplast genome structure of two caffeine-free *C. yungkiangensis* types

**Figure 2:**
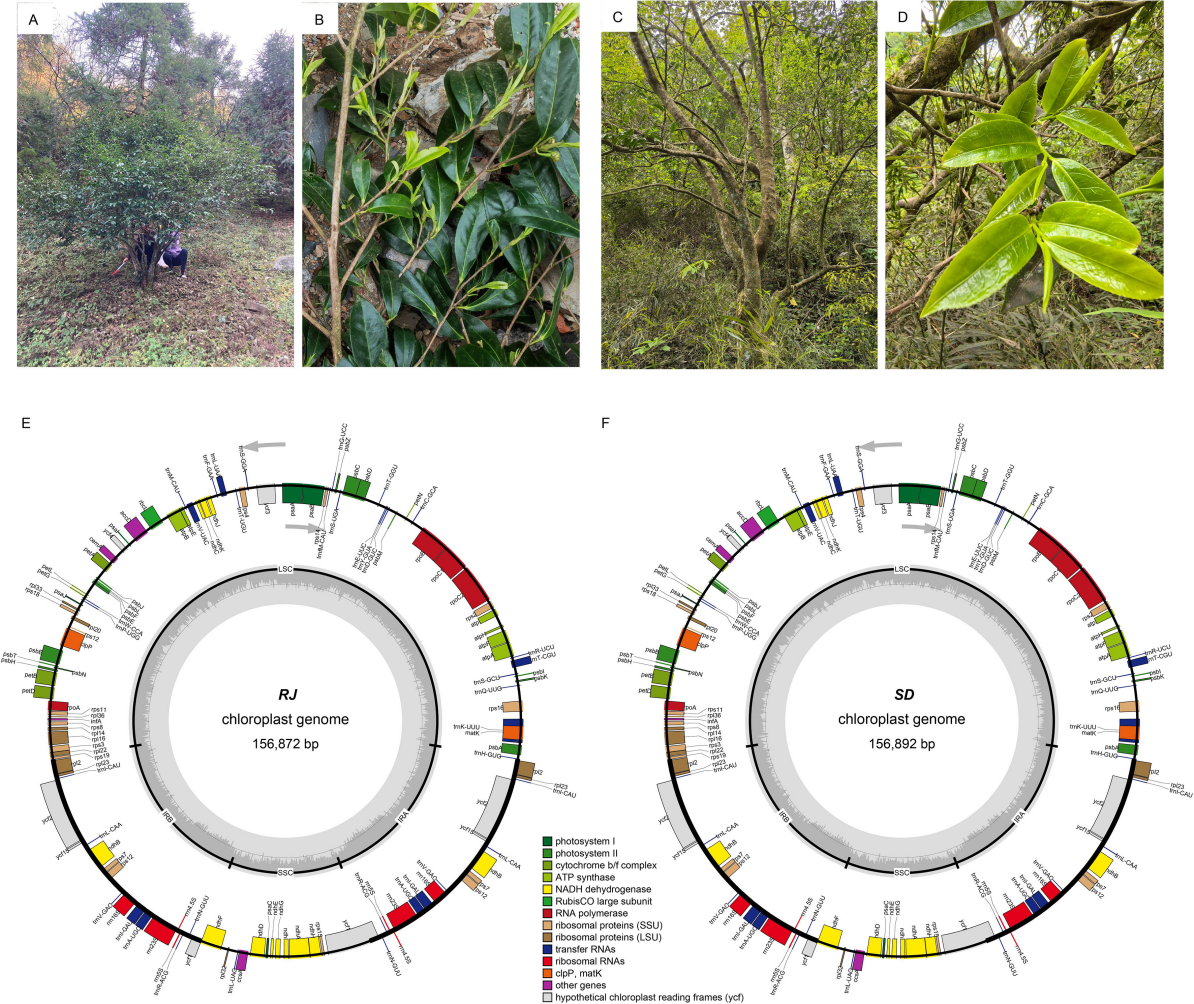
Morphology and chloroplast genome maps of two type of *C. yungkiangensis*. Photographs depict the whole plant **(A)** and branches **(B)** of the RJ specimen. Photographs of the whole plant **(C)** and branches **(D)** of the SD specimen. The schematic map of overall features of the chloroplast genome of RJ **(E)** and SD **(F)**. The arrows indicated the translation directions of inner and outer genes. The inner circle indicates the GC contents.

Based on their tree morphology, these resources were classified into two categories: shrub-type and tree-type. To investigate their phylogenetic relationships with other tea plants (*Camellia* sect. *Thea*) and among themselves, two representative resources—the shrub-type RJ (**Figures 2A, B**) and the tree-type SD (**Figures 2C, D**)—were selected for chloroplast genome sequencing and assembly (**Supplementary Table 1**). The complete chloroplast genomes of both resources were assembled into circular molecules exhibiting a typical quadripartite structure. The RJ chloroplast genome (**Figure 2E**) spans 156,872 bp with an overall GC content of 37.32%. It comprises a LSC region of 86,592 bp, a SSC region of 18,262 bp, and a pair of IRs each 26,009 bp in length (IRA and IRB). The GC contents of the LSC, SSC, and two IRs are 35.32%, 30.57%, and 43.01%, respectively. The SD chloroplast genome (**Figure 2F**) has a total length of 156,892 bp, consisting of an 86,580 bp LSC, an 18,262 bp SSC, and two 26,025 bp IRs. The corresponding GC contents are 37.32% (overall), 35.33% (LSC), 30.57% (SSC), and 42.99% (IR).

Gene annotation was conducted on both chloroplast genomes. The results indicate that each genome was annotated with 133 genes, comprising 80 protein-coding genes (including 8 multi-copy genes), 30 tRNA genes (7 multi-copy), and 4 rRNA genes (all multi-copy). The protein-coding genes encompass 16 functional categories associated with photosynthesis, transcription, translation, and other metabolic processes, including genes encoding NADH dehydrogenase subunits, photosystem I and II subunits, cytochrome b/f complex components, ATP synthase subunits, ribosomal proteins, RuBisCO large subunit, RNA polymerase subunits, maturase, and several conserved open reading frames (**Supplementary Table 2**). Among these, 13 cis-splicing genes including *rps16*, *atpF*, *rpoC1*, *ycf3*, *clpP*, *petB*, *petD*, *rpl16*, *rpl2* (×2), *ndhB* (×2), *ndhA*, and a trans-splicing gene *rps12* were identified (**Supplementary Figure 2**).

### 3.3 Patterns of codon usage bias in protein-coding genes

Codon usage bias analysis was performed on 80 unique protein-coding genes (PCGs) within the chloroplast genomes of RJ and SD. Codons exhibiting a RSCU value exceeding 1.0 were defined as preferentially utilized by their corresponding amino acids. As shown in **Supplementary Figure 3**, with the exception of the start codon (AUG) and the tryptophan codon (UGG), both possessing an RSCU value of 1.0, the majority of chloroplast PCGs demonstrated widespread codon usage bias. For instance, leucine (Leu) exhibited a pronounced preference for the UUA codon, which displayed the highest RSCU value (2.0) among all analyzed PCGs in both chloroplast genomes (**Supplementary Tables 3**, **S4**). In addition, the alanine (Ala) codon GCU and the arginine (Arg) codon AGA exhibited markedly preferential usage (RSCU = 1.84 each) compared to their synonymous counterparts GCG (0.39) and AGG (0.58). These non-random codon usage patterns reflect the influence of specific selective pressures driving the evolution of PCGs in the chloroplast genome (Liu et al., 2025).

### 3.4 Analysis of repetitive elements and simple sequence repeats

**Figure 3:**
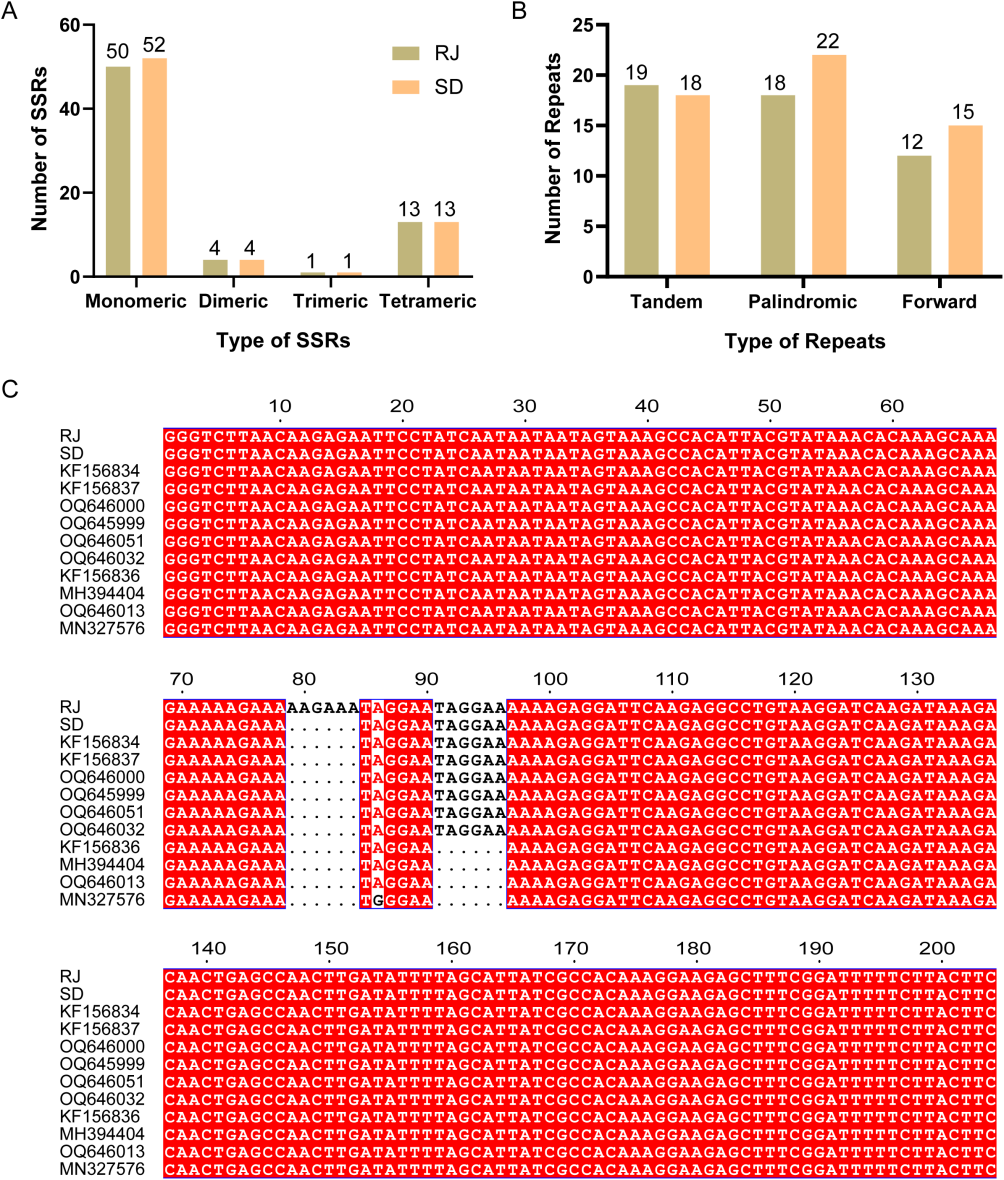
Statistical analysis of repetitive elements. **(A)** Statistics on the types and quantities of SSRs in the RJ and SD chloroplast genomes. **(B)** Statistics on the types and quantities of long repeat sequences in the RJ and SD chloroplast genomes. **(C)** Sequence alignment of different Camellia sect. Thea based on a hexameric SSR on the RJ chloroplast genome.

A comparative analysis of repetitive sequences, including SSRs, tandem repeats, and dispersed repeats, was performed on the chloroplast genomes of RJ and SD. A total of 69 and 70 SSRs were identified in the RJ and SD chloroplast genomes, respectively (**Supplementary Tables 5**, **S6**). Mononucleotide and dinucleotide repeats constituted the predominant types, accounting for 78.26% (RJ) and 80.00% (SD) of all SSRs (**Figure 3A**). Among mononucleotide repeats, thymine (T) repeats were the most abundant, representing 62.00% (31/50) and 61.54% (32/52) in RJ and SD, respectively. Pentameric SSRs were absent in the RJ chloroplast genome, while pentamers and hexamers were not detected in SD. A hexameric SSR, (AAAGAA)_3_, was identified within the RJ chloroplast genome. Sequence alignment indicated its utility for distinguishing RJ from SD (**Figure 3C**).

For tandem repeats, 19 and 18 were identified in the RJ and SD chloroplast genomes, respectively, ranging in length from 12 to 25 bp. Regarding dispersed repeats (length ≥ 30 bp), 30 and 37 pairs were identified in the RJ and SD chloroplast genomes, respectively. These dispersed repeats were exclusively classified as either palindromic or forward repeats. RJ contained 18 palindromic and 12 forward repeat pairs, while SD contains 22 palindromic and 15 forward pairs (**Figure 3B**). No reverse or complementary repeats were detected. The IR region constitutes the longest palindromic repeat pair. The longest forward repeats measured 46 bp in RJ and 64 bp in SD.

### 3.5 Phylogenetic analysis and evolutionary relationships

**Figure 4:**
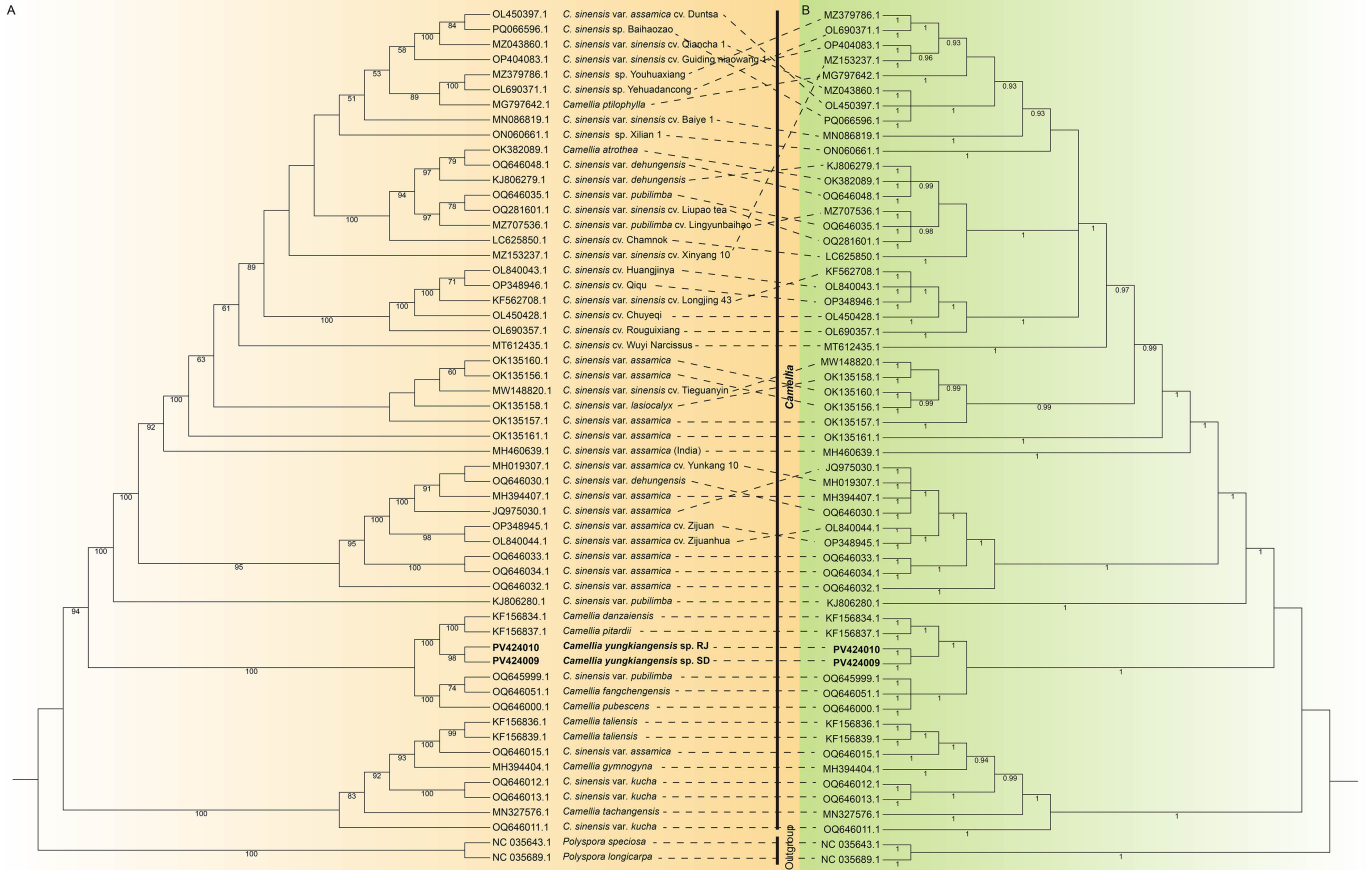
Phylogenetic relationships of different *Camellia* sect. *Thea* species based on their complete chloroplast genome sequences. **(A)** The phylogenetic tree based on the maximum likelihood method. The numbers below each branch are the maximum likelihood bootstrap of each clade >50%. **(B)** The phylogenetic tree based on Bayesian methods. Numbers below the lines indicate the Bayesian posterior probabilities (>0.90).

To investigate the evolutionary relationships between the two types of *C. yungkiangensis* and other species within *Camellia* sect. *Thea*, a phylogenetic tree was reconstructed using their complete chloroplast genome sequences. Phylogenetic analyses were conducted concurrently using two distinct methodologies, yielding highly congruent topologies. As shown in **Figure 4**, a clear differentiation was observed between the two outgroup species and the members of sect. *Thea*. These two caffeine-free germplasms (RJ and SD) formed a distinct clade, indicative of their close evolutionary relationship and reflecting their divergence from other species within sect. *Thea*. This finding aligns with prior results derived from nuclear genome analyses (Guo et al., 2021). In addition, the results demonstrated that RJ and SD share a common clade with *C. pitardii* and *C. danzaiensis*, suggesting a close evolutionary affinity among these taxa. Notably, within Ming’s taxonomic system, both *C. yungkiangensis* and *C. danzaiensis* are categorized under *C. costata* (Ming, 1992). Our findings provide novel genetic-level evidence supporting this relationship. However, the phylogenetic tree also revealed unexpected relationships. For instance, while nuclear genome analyses identified clear divergence between CSS and CSA (Tong et al., 2025; Kong et al., 2025), chloroplast genome analyses indicated that they do not form entirely distinct clades. This phenomenon has been documented in previous studies (Li et al., 2021; Liu et al., 2022; Qiao, 2024) and may be attributed to inherent characteristics of sect. *Thea*. The section encompasses a vast number of species, yet current classification relies predominantly on phenotypic traits (Zhao, 2024). Furthermore, widespread natural hybridization among its species complicates phenotype-based classification accuracy. This also underscores the limitations of relying exclusively on the chloroplast genome for reconstructing the phylogeny of sect. *Thea* (Qiao, 2024).

Based on the phylogenetic tree, other sect. *Thea* plants closely related to RJ and SD, including *C. danzaiensis* (KF156834), *C. pitardii* (KF156837), *C. pubescens* (OQ646000), *C. sinensis* var. *pubilimba* (OQ645999), *C. fangchengensis* (OQ646051), *C. sinensis* var. *assamica* (OQ646032), *C. taliensis* (KF156836), *C. gymnogyna* (MH394404), *C. sinensis* var. *kucha* (OQ646013), and *C. tachangensis* (MN327576) were selected for sequence alignment. The results indicated that the hexameric SSR in RJ seems to be specific to it. This molecular marker can differentiate RJ not only from SD but also from all other examined plants within the section (**Figure 3C**). This provides the potential for developing species-specific molecular markers using the chloroplast genome.

### 3.6 Comparative analysis of IR region boundaries

To investigate the evolutionary dynamics of chloroplast genomes in the genus *Camellia*, we selected 11 chloroplast genomes for comparative analysis based on phylogenetic tree results. These encompassed cultivated tea plants and several of its varieties, as along with closely related species. The IRscope was employed to visualize the boundaries of the LSC, SSC, and IR regions, as well as adjacent genes. As shown in **Supplementary Figure 4**, all analyzed chloroplast genomes exhibited a conserved quadripartite structure, comprising an LSC region, an SSC region, and a pair of IR regions. Total genome length ranged from 156,762 bp (MT612435.1) to 157,353 bp (MH460639.1). The length of the IR regions was also highly stable, ranging from approximately 26,009 bp (RJ) to 26,095 bp (MW148820.1).

The structure of the JLB junction (LSC/IRb boundary) was highly conserved across all samples. The gene arrangement flanking this boundary was identical: the *rpl22* gene resided entirely within the LSC region, while the *rps19* gene spanned the JLB boundary. Specifically, the 5’ end of the *rps19* gene initiated within the LSC region, and its 3’ end extended into the IRb region, forming a characteristic boundary-spanning structure. Among these 11 genomes, the rps19 gene spanned 46 bp into the IRb region, except for MH460639.1, which exhibited a 2-bp overlap. This precise gene arrangement pattern indicates that the JLB boundary is a highly conserved region in the chloroplast genome evolution of *Camellia* species. Interestingly, MH460639.1 represents the sole CSA tea plant originating from India within this sample set, suggesting it may have undergone distinct evolutionary pathways. This phenomenon has also been observed in earlier studies (Li et al., 2021). Our expanded sample size comparison further revealed differences in chloroplast genomes structure between Chinese and Indian tea accessions. The boundary between the SSC region and the two flanking IR regions (JSB and JSA) exhibited the most significant variation observed in this study. Both boundaries are spanned by the pseudogene *ycf1*, and the sliding of these boundaries constitutes the primary factor contributing to fluctuations in SSC region length and variations in total genome size. Notably, we found that SD and RJ exhibited identical boundary sliding lengths at both JSB and JSA compared to *C. sinensis* var. *pubilimba* (OQ645999.1).

### 3.7 Colinearity and nucleotide diversity analysis

Furthermore, colinearity analysis of these chloroplast genomes was performed using the Mauve software. The results indicated an absence of rearrangements or inversions (**Supplementary Figure 5A**), demonstrating remarkable conservation of chloroplast genomes across different sect. *Thea* plants.

Multiple sequence alignment and subsequent nucleotide diversity analysis revealed non-uniform sequence conservation across the genome, with a pronounced disparity observed between single-copy versus repeat regions (**Supplementary Figure 5B**). The genome-wide nucleotide diversity (Pi) ranged from 0 to 0.0108, with a mean of 0.0014. Ten genes exhibiting Pi > 0.006 were identified, ranked in descending order: *trnH-psbA, matK, ndhF-rpl32, psaA-ycf3, trnT, rps3, ndhF, rpl32-trnL, petN-psbM*, and *ycf1*. They were all located within the single-copy regions, indicating that the LSC and SSC regions exhibit greater sequence diversity compared to the IR region.

## 4 Conclusion

In this study, we determined that the caffeine-free trait is a consistent and stable attribute of *C. yungkiangensis*, which is unaffected by either geographical translocation or cultivation practices. Subsequently, the complete chloroplast genomes of both the shrub (RJ) and tree (SD) forms of *C. yungkiangensis* were sequenced and characterized. The chloroplast genome structures and gene contents of both forms were similar to those reported in other species of sect. *Thea*. Repetitive sequence analysis revealed that a hexameric SSR marker could distinguish between the two forms. Phylogenetic analysis showed that they formed a monophyletic clade, exhibiting close phylogenetic relationships with *C. pitardii* and *C. danzaiensis*. This study provides new insights into the phylogenetic evolution of sect. *Thea* and contributes to the conservation and utilization of these valuable germplasm resources.

## Data Availability

The datasets presented in this study can be found in online repositories. The names of the repository/repositories and accession number(s) can be found in the article/**Supplementary Material**.
