# Supplementary material for: Complete chloroplast genome characterization and phylogenetic analysis of two natural caffeine-free Camellia yungkiangensis H. T. Chang accessions
Source: Front Plant Sci. 2026 Apr 28;17:1807875. doi: 10.3389/fpls.2026.1807875 (PMC13161175; doi:10.3389/fpls.2026.1807875)
Supplement: Supplementary file 1 [file DataSheet1.docx]

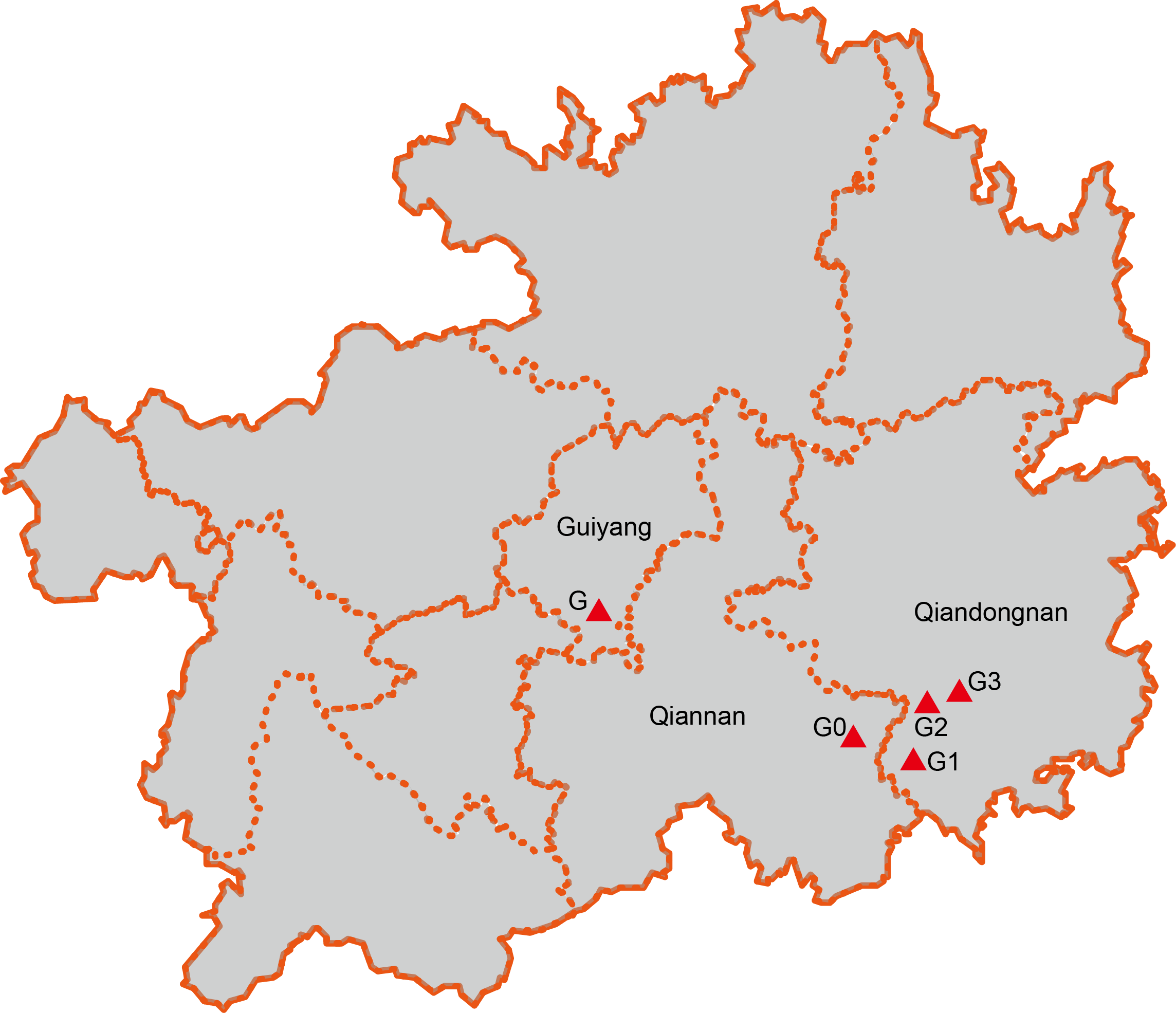


**Figure S1**. The locations of the sampling sites in this study on the map.


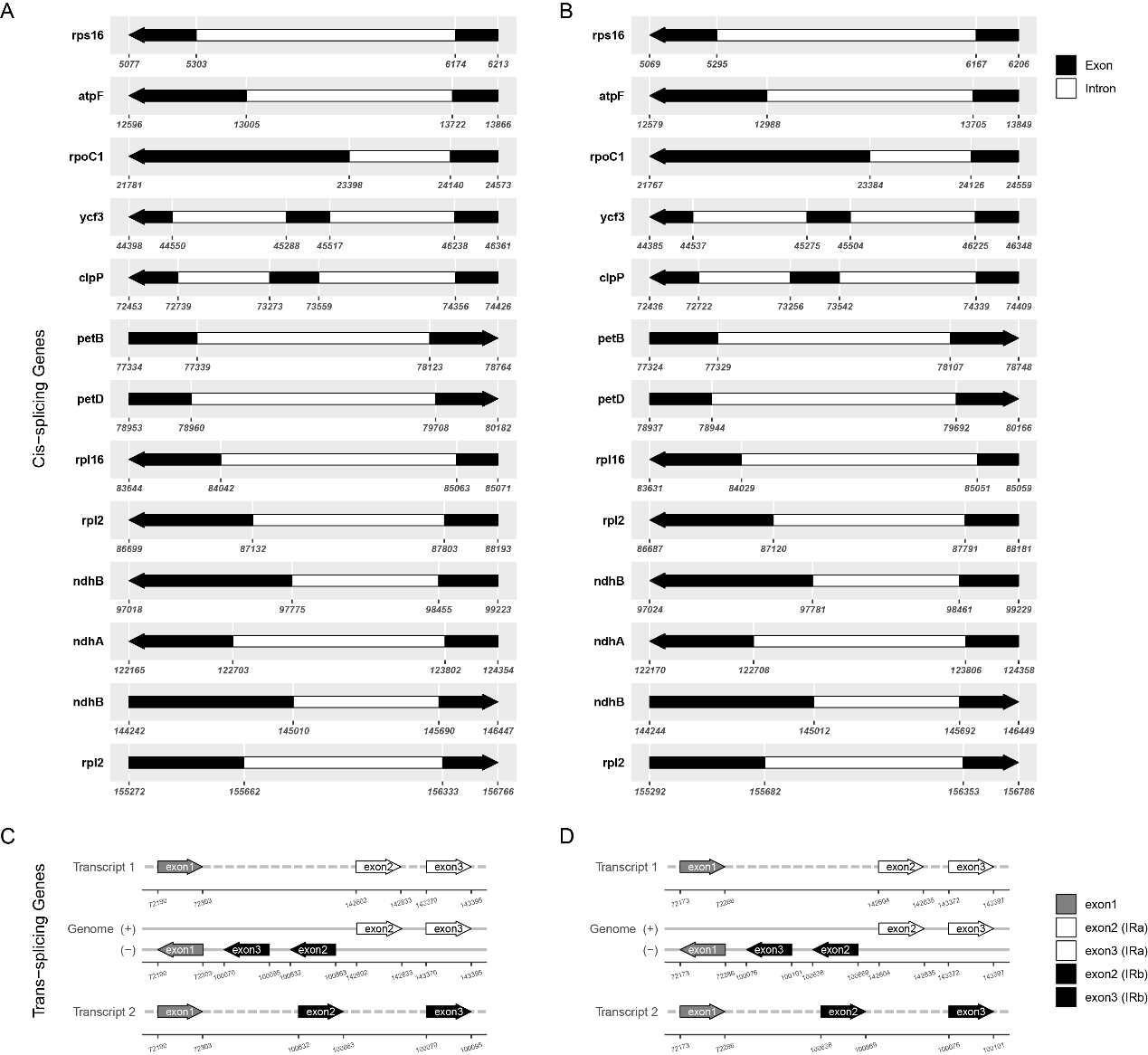


**Figure S2**. The splicing genes identified from the chloroplast genomes of RJ and SD by using CPGview. (A-B) Schematic map of the cis-splicing genes. The genes are arranged from top to bottom based on their order on the cp genome. The gene names are shown on the left, and the gene structures are on the right. The exons are shown in black and the introns are shown in white. The arrow indicates the sense direction of the gene. (C-D) Schematic map of the trans-splicing gene rps12.


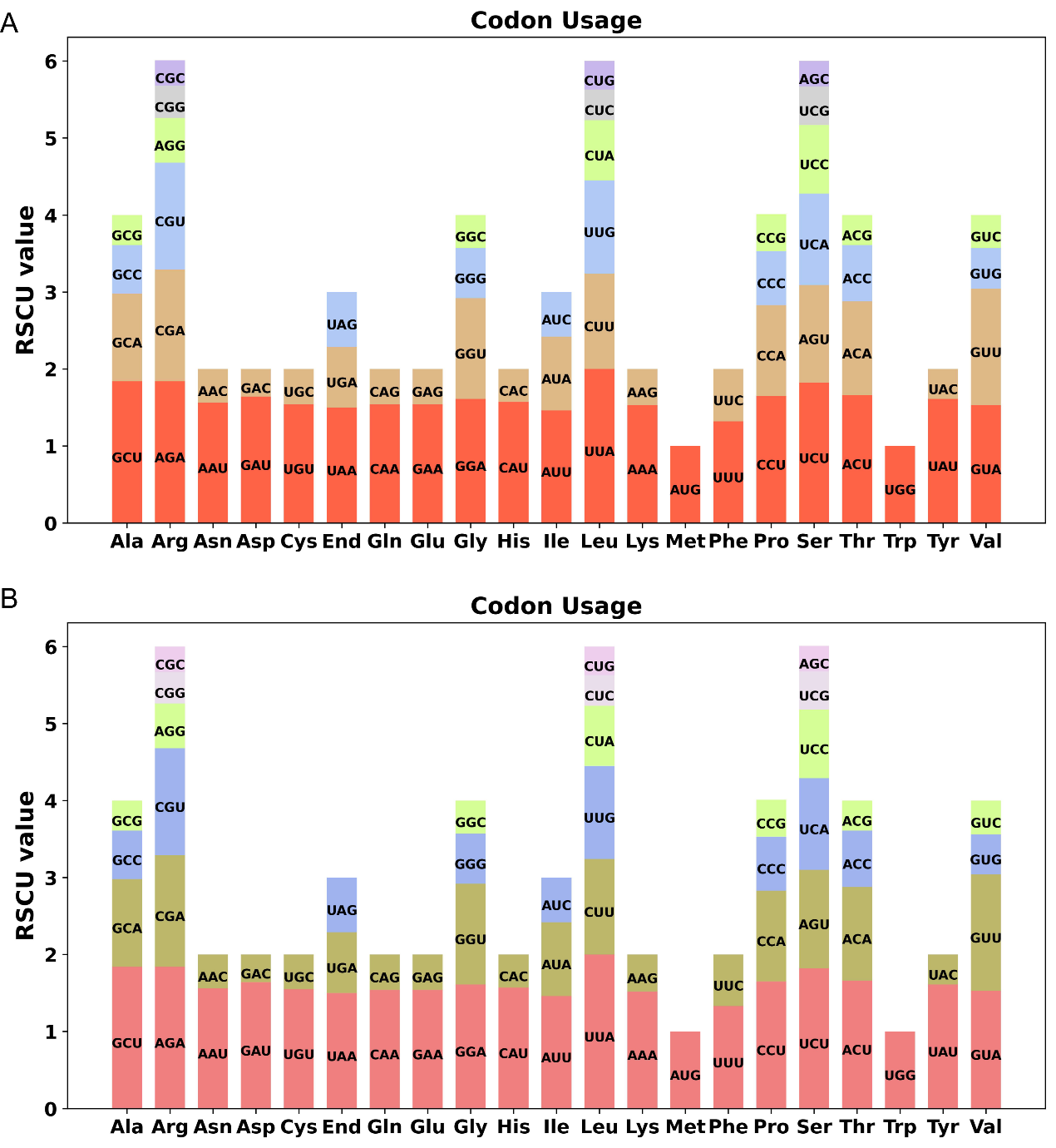


**Figure S3**. Statistical comparison of relative synonymous codon usage (RSCU) of chloroplast genes in RJ (A) and SD (B).


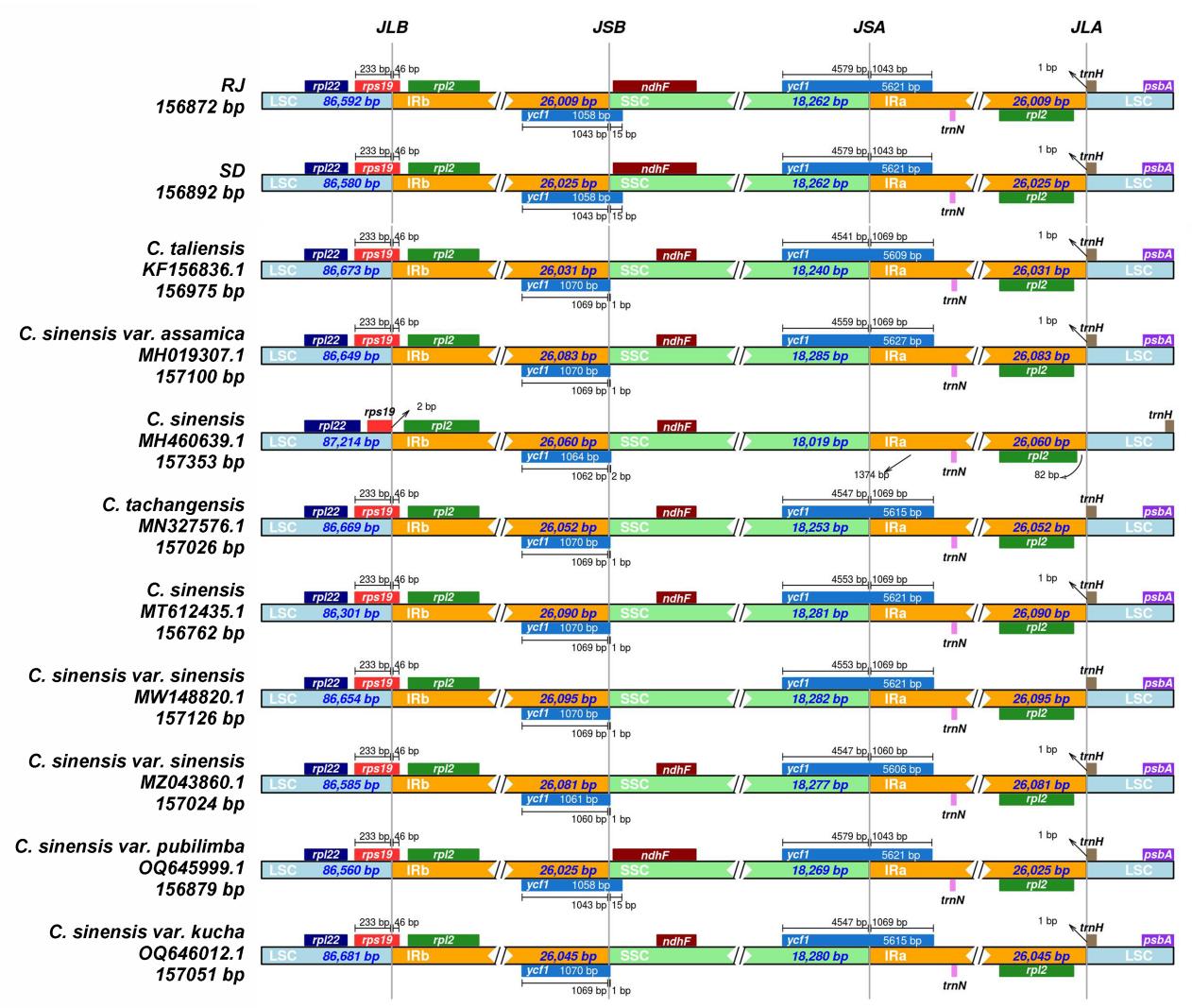


**Figure S4**. Structural comparison of IR boundaries across eleven *Camellia* genus chloroplast genomes. The diagram illustrates four key junction points: LSC/IRb (JLB), IRb/SSC (JSB), SSC/IRa (JSA), and IRa/LSC (JLA). Numbers within boxes indicate the distance (in base pairs) between gene terminals and boundary positions.


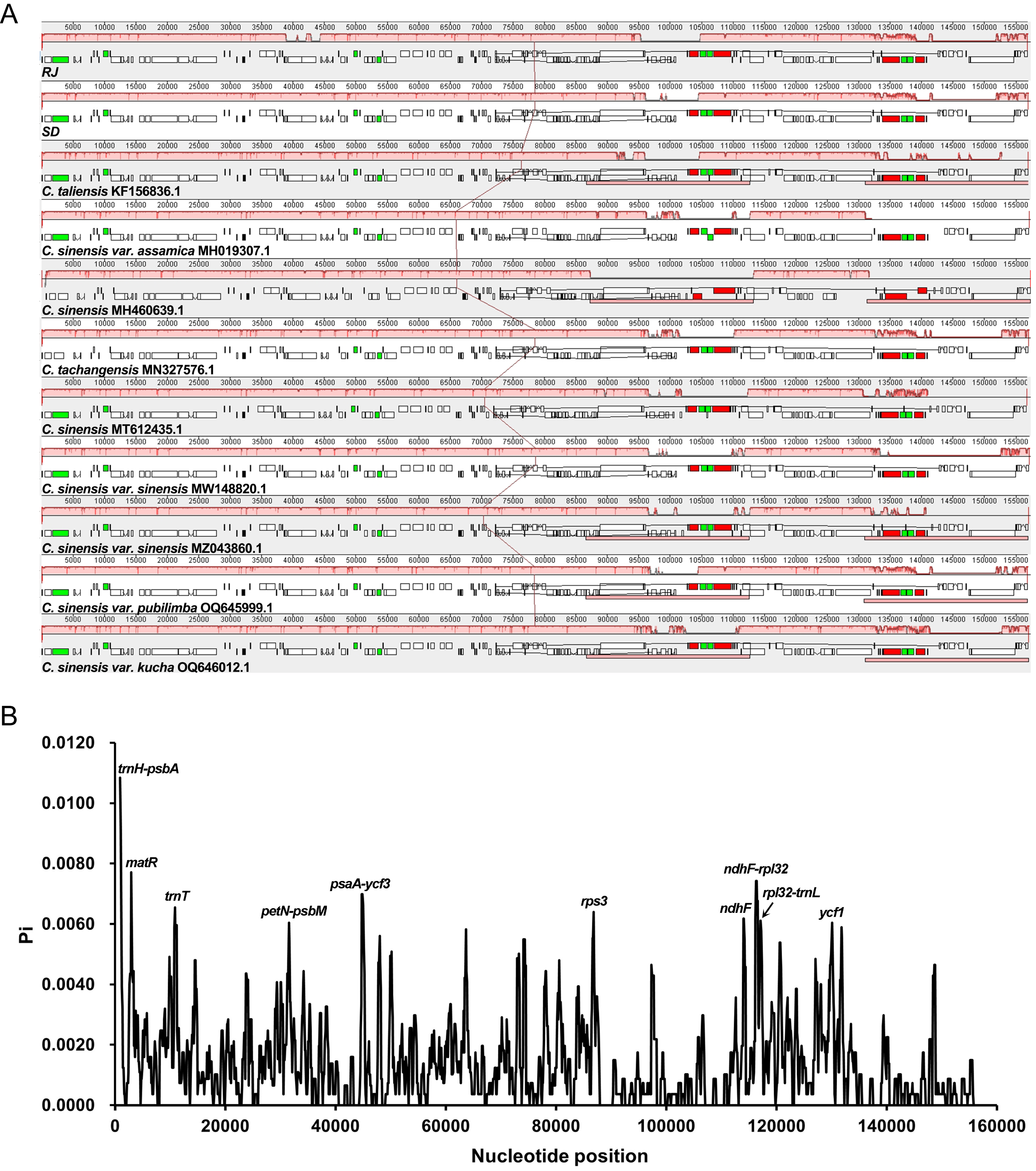


**Figure S5**. Colinearity and nucleotide diversity analysis across eleven sect*. Thea* plant chloroplast genomes. (A) Collinearity analysis of the chloroplast genomes from eleven sect*. Thea* plants. Blocks sharing identical colors represent homologous regions. Homologous blocks are interconnected by lines, with each block illustrating the trend of sequence similarity within its constituent fragments. White rectangles represent CDS, red rectangles denote rRNA, and green rectangles denote tRNA. Introns are connected by lines. (B) Nucleotide diversity analysis of the eleven sect*. Thea* plant chloroplast genomes. The x-axis represents the position along the aligned sequences, while the y-axis displays nucleotide diversity values. Each data point represents to the nucleotide diversity calculated per 100 bp window.
