# Supplementary material for: Complete chloroplast genome characterization and phylogenetic analysis of two natural caffeine-free Camellia yungkiangensis H. T. Chang accessions
Source: Front Plant Sci. 2026 Apr 28;17:1807875. doi: 10.3389/fpls.2026.1807875 (PMC13161175; doi:10.3389/fpls.2026.1807875)
Supplement: Supplementary file 3 [file Table2.docx]

**Table S2**. Gene composition of the chloroplast genomes from two caffeine-free tea plant resources, RJ and SD. The number in parentheses represents the copy number of the gene.

| **Group of genes** | **Name of genes** |
| --- | --- |
| Subunits of NADH-dehydrogenase | *ndh*A, *ndh*B(×2), *ndh*C, *ndh*D, *ndh*E, *ndh*F, *ndh*G, *ndh*H, *ndh*I, *ndh*J, *ndh*K |
| Subunits of photosystem I | *psa*A, *psa*B, *psa*C, *psa*I, *psa*J |
| Subunits of photosystem II | *psb*A, *psb*B, *psb*C, *psb*D, *psb*E, *psb*F, *psb*H, *psb*I, *psb*J, *psb*K, *psb*L, *psb*M, *psb*N, *psb*T, *psb*Z, *ycf*3 |
| Subunits of cytochrome b/f complex | *pet*A, *pet*B, *pet*D, *pet*G, *pet*L, *pet*N |
| Subunits of ATP synthase | *atp*A, *atp*B, *atp*E, *atp*F, *atp*H, *atp*I |
| Large subunit of rubisco | *rbc*L |
| Small subunit of ribosome | *rps*2, *rps*3, *rps*4, *rps*7(×2), *rps*8, *rps*11, *rps*12(×2), *rps*14, *rps*15, *rps*16, *rps*18, *rps*19 |
| Large subunit of ribosome | *rpl*2(×2), *rpl*14, *rpl*16, *rpl*20, *rpl*22, *rpl*23(×2), *rpl*32, *rpl*33, *rpl*36 |
| DNA dependent RNA polymerase | *rpo*A, *rpo*B, *rpo*C1, *rpo*C2 |
| rRNA genes | *rrn*4.5S(×2), *rrn*5S(×2), *rrn*16S(×2), *rrn*23S(×2) |
| tRNA genes | *trn*A-UGC(×2), *trn*C-GCA, *trn*D-GUC, *trn*E-UUC, *trn*F-GAA, *trn*fM-CAU, *trn*G-UCC, *trn*H-GUG, *trn*I-CAU(×2), *trn*I-GAU(×2), *trn*K-UUU, *trn*L-CAA(×2), *trn*L-UAA, *trn*L-UAG, *trn*M-CAU, *trn*N-GUU(×2), *trn*P-UGG, trnQ-UUG, *trn*R-ACG(×2), *trn*R-UCU, *trn*S-GCU, *trn*S-GGA, *trn*S-UGA, *trn*T-CGU, *trn*T-GGU, *trn*T-UGU, *trn*V-GAC(×2), *trn*V-UAC, *trn*W-CCA, *trn*Y-GUA |
| Maturase | *mat*K |
| c-type cytochrom synthesis gene | *ccs*A |
| Envelope membrane protein | *cem*A |
| Protease | *clp*P |
| Subunit of acetyl-CoA-carboxylase | *acc*D |
| Translational initiation factor | *inf*A |
| Genes of unknown functions open reading | *ycf*1(×2), *ycf*2(×2), *ycf*4, *ycf*15(×2) |

**Table S3**. Relative synonymous codon usage (RSCU) for each amino acid in the RJ chloroplast genome.

| Amino acid | Codon 1 | Codon 2 | Codon 3 | Codon 4 | Codon 5 | Codon 6 |
| --- | --- | --- | --- | --- | --- | --- |
|  | RSCU | RSCU | RSCU | RSCU | RSCU | RSCU |
| Ala | GCU | GCA | GCC | GCG |  |  |
|  | 1.84 | 1.14 | 0.63 | 0.39 |  |  |
| Arg | AGA | CGA | CGU | AGG | CGG | CGC |
|  | 1.84 | 1.45 | 1.39 | 0.58 | 0.42 | 0.33 |
| Asn | AAU | AAC |  |  |  |  |
|  | 1.56 | 0.44 |  |  |  |  |
| Asp | GAU | GAC |  |  |  |  |
|  | 1.64 | 0.36 |  |  |  |  |
| Cys | UGU | UGC |  |  |  |  |
|  | 1.54 | 0.46 |  |  |  |  |
| End | UAA | UGA | UAG |  |  |  |
|  | 1.5 | 0.79 | 0.71 |  |  |  |
| Gln | CAA | CAG |  |  |  |  |
|  | 1.54 | 0.46 |  |  |  |  |
| Glu | GAA | GAG |  |  |  |  |
|  | 1.54 | 0.46 |  |  |  |  |
| Gly | GGA | GGU | GGG | GGC |  |  |
|  | 1.61 | 1.31 | 0.65 | 0.43 |  |  |
| His | CAU | CAC |  |  |  |  |
|  | 1.57 | 0.43 |  |  |  |  |
| Ile | AUU | AUA | AUC |  |  |  |
|  | 1.46 | 0.96 | 0.58 |  |  |  |
| Leu | UUA | CUU | UUG | CUA | CUC | CUG |
|  | 2 | 1.24 | 1.21 | 0.78 | 0.4 | 0.37 |
| Lys | AAA | AAG |  |  |  |  |
|  | 1.53 | 0.47 |  |  |  |  |
| Met | AUG |  |  |  |  |  |
|  | 1 |  |  |  |  |  |
| Phe | UUU | UUC |  |  |  |  |
|  | 1.32 | 0.68 |  |  |  |  |
| Pro | CCU | CCA | CCC | CCG |  |  |
|  | 1.65 | 1.18 | 0.7 | 0.48 |  |  |
| Ser | UCU | AGU | UCA | UCC | UCG | AGC |
|  | 1.82 | 1.27 | 1.19 | 0.89 | 0.5 | 0.33 |
| Thr | ACU | ACA | ACC | ACG |  |  |
|  | 1.66 | 1.22 | 0.73 | 0.39 |  |  |
| Trp | UGG |  |  |  |  |  |
|  | 1 |  |  |  |  |  |
| Tyr | UAU | UAC |  |  |  |  |
|  | 1.61 | 0.39 |  |  |  |  |
| Val | GUA | GUU | GUG | GUC |  |  |
|  | 1.53 | 1.51 | 0.53 | 0.43 |  |  |

**Table S4**. Relative synonymous codon usage (RSCU) for each amino acid in the SD chloroplast genome.

| Amino acid | Codon 1 | Codon 2 | Codon 3 | Codon 4 | Codon 5 | Codon 6 |
| --- | --- | --- | --- | --- | --- | --- |
|  | RSCU | RSCU | RSCU | RSCU | RSCU | RSCU |
| Ala | GCU | GCA | GCC | GCG |  |  |
|  | 1.84 | 1.14 | 0.63 | 0.39 |  |  |
| Arg | AGA | CGA | CGU | AGG | CGG | CGC |
|  | 1.84 | 1.45 | 1.39 | 0.58 | 0.41 | 0.33 |
| Asn | AAU | AAC |  |  |  |  |
|  | 1.56 | 0.44 |  |  |  |  |
| Asp | GAU | GAC |  |  |  |  |
|  | 1.64 | 0.36 |  |  |  |  |
| Cys | UGU | UGC |  |  |  |  |
|  | 1.55 | 0.45 |  |  |  |  |
| End | UAA | UGA | UAG |  |  |  |
|  | 1.5 | 0.79 | 0.71 |  |  |  |
| Gln | CAA | CAG |  |  |  |  |
|  | 1.54 | 0.46 |  |  |  |  |
| Glu | GAA | GAG |  |  |  |  |
|  | 1.54 | 0.46 |  |  |  |  |
| Gly | GGA | GGU | GGG | GGC |  |  |
|  | 1.61 | 1.31 | 0.65 | 0.43 |  |  |
| His | CAU | CAC |  |  |  |  |
|  | 1.57 | 0.43 |  |  |  |  |
| Ile | AUU | AUA | AUC |  |  |  |
|  | 1.46 | 0.96 | 0.58 |  |  |  |
| Leu | UUA | CUU | UUG | CUA | CUC | CUG |
|  | 2 | 1.24 | 1.21 | 0.78 | 0.4 | 0.37 |
| Lys | AAA | AAG |  |  |  |  |
|  | 1.52 | 0.48 |  |  |  |  |
| Met | AUG |  |  |  |  |  |
|  | 1 |  |  |  |  |  |
| Phe | UUU | UUC |  |  |  |  |
|  | 1.33 | 0.67 |  |  |  |  |
| Pro | CCU | CCA | CCC | CCG |  |  |
|  | 1.65 | 1.18 | 0.7 | 0.48 |  |  |
| Ser | UCU | AGU | UCA | UCC | UCG | AGC |
|  | 1.82 | 1.28 | 1.19 | 0.89 | 0.5 | 0.33 |
| Thr | ACU | ACA | ACC | ACG |  |  |
|  | 1.66 | 1.22 | 0.73 | 0.39 |  |  |
| Trp | UGG |  |  |  |  |  |
|  | 1 |  |  |  |  |  |
| Tyr | UAU | UAC |  |  |  |  |
|  | 1.61 | 0.39 |  |  |  |  |
| Val | GUA | GUU | GUG | GUC |  |  |
|  | 1.53 | 1.51 | 0.52 | 0.44 |  |  |

| **Table S5**. SSRs in the chloroplast genome of RJ. | | | | | | |
| --- | --- | --- | --- | --- | --- | --- |
| Chloroplast genome | SSR nr. | SSR type | SSR | size | start | end |
| RJ | 1 | p1 | (A)11 | 11 | 365 | 375 |
| RJ | 2 | p4 | (AGAT)3 | 12 | 6726 | 6737 |
| RJ | 3 | p1 | (T)11 | 11 | 8774 | 8784 |
| RJ | 4 | p1 | (A)13 | 13 | 8917 | 8929 |
| RJ | 5 | p1 | (C)10 | 10 | 10973 | 10982 |
| RJ | 6 | p4 | (GTCT)3 | 12 | 12015 | 12026 |
| RJ | 7 | p1 | (A)17 | 17 | 12559 | 12575 |
| RJ | 8 | p1 | (T)13 | 13 | 13932 | 13944 |
| RJ | 9 | p1 | (T)11 | 11 | 15059 | 15069 |
| RJ | 10 | p1 | (T)13 | 13 | 15458 | 15470 |
| RJ | 11 | p1 | (A)11 | 11 | 17279 | 17289 |
| RJ | 12 | p1 | (T)11 | 11 | 19473 | 19483 |
| RJ | 13 | p2 | (AT)5 | 10 | 20840 | 20849 |
| RJ | 14 | p1 | (T)10 | 10 | 27167 | 27176 |
| RJ | 15 | p1 | (A)11 | 11 | 32910 | 32920 |
| RJ | 16 | p1 | (T)17 | 17 | 33214 | 33230 |
| RJ | 17 | p1 | (T)12 | 12 | 34004 | 34015 |
| RJ | 18 | p4 | (TCTT)3 | 12 | 34103 | 34114 |
| RJ | 19 | p1 | (A)13 | 13 | 37615 | 37627 |
| RJ | 20 | p1 | (A)10 | 10 | 38255 | 38264 |
| RJ | 21 | p1 | (A)12 | 12 | 38487 | 38498 |
| RJ | 22 | p4 | (TTTC)3 | 12 | 45244 | 45255 |
| RJ | 23 | p1 | (A)12 | 12 | 46212 | 46223 |
| RJ | 24 | p1 | (A)11 | 11 | 46468 | 46478 |
| RJ | 25 | p4 | (AAAG)3 | 12 | 46476 | 46487 |
| RJ | 26 | p1 | (T)10 | 10 | 48903 | 48912 |
| RJ | 27 | p1 | (A)12 | 12 | 49315 | 49326 |
| RJ | 28 | p1 | (T)11 | 11 | 49972 | 49982 |
| RJ | 29 | p1 | (T)14 | 14 | 53340 | 53353 |
| RJ | 30 | p1 | (T)10 | 10 | 56302 | 56311 |
| RJ | 31 | p1 | (T)15 | 15 | 56736 | 56750 |
| RJ | 32 | p1 | (T)11 | 11 | 58953 | 58963 |
| RJ | 33 | p1 | (T)14 | 14 | 60731 | 60744 |
| RJ | 34 | p4 | (AAAT)3 | 12 | 62662 | 62673 |
| RJ | 35 | p1 | (A)11 | 11 | 65278 | 65288 |
| RJ | 36 | p1 | (T)10 | 10 | 65417 | 65426 |
| RJ | 37 | p1 | (T)12 | 12 | 65733 | 65744 |
| RJ | 38 | p3 | (TTC)4 | 12 | 70056 | 70067 |
| RJ | 39 | p1 | (T)10 | 10 | 70934 | 70943 |
| RJ | 40 | p6 | (AAAGAA)3 | 18 | 77826 | 77843 |
| RJ | 41 | p1 | (T)10 | 10 | 80602 | 80611 |
| RJ | 42 | p1 | (T)15 | 15 | 82518 | 82532 |
| RJ | 43 | p1 | (T)15 | 15 | 83048 | 83062 |
| RJ | 44 | p1 | (A)10 | 10 | 83570 | 83579 |
| RJ | 45 | p2 | (AT)5 | 10 | 84282 | 84291 |
| RJ | 46 | p1 | (T)10 | 10 | 85019 | 85028 |
| RJ | 47 | p2 | (TA)5 | 10 | 87249 | 87258 |
| RJ | 48 | p4 | (TCTA)3 | 12 | 94586 | 94597 |
| RJ | 49 | p1 | (T)10 | 10 | 105256 | 105265 |
| RJ | 50 | p4 | (CCCT)3 | 12 | 110014 | 110025 |
| RJ | 51 | p1 | (A)12 | 12 | 110314 | 110325 |
| RJ | 52 | p1 | (T)13 | 13 | 115250 | 115262 |
| RJ | 53 | p1 | (A)11 | 11 | 117061 | 117071 |
| RJ | 54 | p1 | (T)10 | 10 | 117883 | 117892 |
| RJ | 55 | p4 | (GAAA)3 | 12 | 118048 | 118059 |
| RJ | 56 | p4 | (AATA)3 | 12 | 118198 | 118209 |
| RJ | 57 | p4 | (AAAT)3 | 12 | 121192 | 121203 |
| RJ | 58 | p1 | (T)14 | 14 | 121254 | 121267 |
| RJ | 59 | p1 | (T)10 | 10 | 122962 | 122971 |
| RJ | 60 | p1 | (T)12 | 12 | 127235 | 127246 |
| RJ | 61 | p1 | (T)12 | 12 | 128850 | 128861 |
| RJ | 62 | p1 | (A)12 | 12 | 129154 | 129165 |
| RJ | 63 | p1 | (A)10 | 10 | 129687 | 129696 |
| RJ | 64 | p1 | (T)17 | 17 | 130068 | 130084 |
| RJ | 65 | p1 | (T)12 | 12 | 133140 | 133151 |
| RJ | 66 | p4 | (GAGG)3 | 12 | 133439 | 133450 |
| RJ | 67 | p1 | (A)10 | 10 | 138200 | 138209 |
| RJ | 68 | p4 | (ATAG)3 | 12 | 148867 | 148878 |
| RJ | 69 | p2 | (AT)5 | 10 | 156206 | 156215 |

| **Table S6**. SSRs in the chloroplast genome of SD. | | | | | | |
| --- | --- | --- | --- | --- | --- | --- |
| Chloroplast genome | SSR nr. | SSR type | SSR | size | start | end |
| SD | 1 | p1 | (A)11 | 11 | 356 | 366 |
| SD | 2 | p1 | (A)10 | 10 | 3789 | 3798 |
| SD | 3 | p1 | (A)10 | 10 | 5501 | 5510 |
| SD | 4 | p4 | (AGAT)3 | 12 | 6719 | 6730 |
| SD | 5 | p1 | (T)11 | 11 | 8767 | 8777 |
| SD | 6 | p1 | (A)11 | 11 | 8910 | 8920 |
| SD | 7 | p4 | (GTCT)3 | 12 | 11999 | 12010 |
| SD | 8 | p1 | (A)16 | 16 | 12543 | 12558 |
| SD | 9 | p1 | (T)15 | 15 | 13915 | 13929 |
| SD | 10 | p1 | (T)12 | 12 | 15044 | 15055 |
| SD | 11 | p1 | (T)13 | 13 | 15444 | 15456 |
| SD | 12 | p1 | (A)11 | 11 | 17265 | 17275 |
| SD | 13 | p1 | (T)11 | 11 | 19459 | 19469 |
| SD | 14 | p2 | (AT)5 | 10 | 20826 | 20835 |
| SD | 15 | p1 | (T)10 | 10 | 27153 | 27162 |
| SD | 16 | p1 | (A)10 | 10 | 32896 | 32905 |
| SD | 17 | p1 | (T)16 | 16 | 33199 | 33214 |
| SD | 18 | p1 | (T)10 | 10 | 33558 | 33567 |
| SD | 19 | p1 | (T)13 | 13 | 33989 | 34001 |
| SD | 20 | p4 | (TCTT)3 | 12 | 34089 | 34100 |
| SD | 21 | p1 | (A)13 | 13 | 37601 | 37613 |
| SD | 22 | p1 | (A)11 | 11 | 38242 | 38252 |
| SD | 23 | p1 | (A)11 | 11 | 38475 | 38485 |
| SD | 24 | p4 | (TTTC)3 | 12 | 45231 | 45242 |
| SD | 25 | p1 | (A)12 | 12 | 46199 | 46210 |
| SD | 26 | p1 | (A)11 | 11 | 46455 | 46465 |
| SD | 27 | p4 | (AAAG)3 | 12 | 46463 | 46474 |
| SD | 28 | p1 | (T)10 | 10 | 48890 | 48899 |
| SD | 29 | p1 | (A)12 | 12 | 49302 | 49313 |
| SD | 30 | p1 | (T)11 | 11 | 49959 | 49969 |
| SD | 31 | p1 | (T)11 | 11 | 52598 | 52608 |
| SD | 32 | p1 | (T)14 | 14 | 53329 | 53342 |
| SD | 33 | p1 | (T)10 | 10 | 56291 | 56300 |
| SD | 34 | p1 | (T)14 | 14 | 56725 | 56738 |
| SD | 35 | p1 | (T)11 | 11 | 58941 | 58951 |
| SD | 36 | p1 | (T)14 | 14 | 60719 | 60732 |
| SD | 37 | p4 | (AAAT)3 | 12 | 62641 | 62652 |
| SD | 38 | p1 | (A)10 | 10 | 65257 | 65266 |
| SD | 39 | p1 | (T)10 | 10 | 65395 | 65404 |
| SD | 40 | p1 | (T)11 | 11 | 65711 | 65721 |
| SD | 41 | p3 | (TTC)4 | 12 | 70033 | 70044 |
| SD | 42 | p1 | (T)10 | 10 | 70911 | 70920 |
| SD | 43 | p1 | (T)10 | 10 | 80586 | 80595 |
| SD | 44 | p1 | (T)10 | 10 | 82508 | 82517 |
| SD | 45 | p1 | (T)16 | 16 | 83033 | 83048 |
| SD | 46 | p1 | (A)11 | 11 | 83556 | 83566 |
| SD | 47 | p2 | (AT)5 | 10 | 84269 | 84278 |
| SD | 48 | p1 | (T)11 | 11 | 85006 | 85016 |
| SD | 49 | p2 | (TA)5 | 10 | 87237 | 87246 |
| SD | 50 | p4 | (TCTA)3 | 12 | 94592 | 94603 |
| SD | 51 | p1 | (T)10 | 10 | 105260 | 105269 |
| SD | 52 | p4 | (CCCT)3 | 12 | 110018 | 110029 |
| SD | 53 | p1 | (A)12 | 12 | 110318 | 110329 |
| SD | 54 | p1 | (T)12 | 12 | 115254 | 115265 |
| SD | 55 | p1 | (A)11 | 11 | 117065 | 117075 |
| SD | 56 | p1 | (T)10 | 10 | 117887 | 117896 |
| SD | 57 | p4 | (GAAA)3 | 12 | 118052 | 118063 |
| SD | 58 | p4 | (AATA)3 | 12 | 118202 | 118213 |
| SD | 59 | p4 | (AAAT)3 | 12 | 121197 | 121208 |
| SD | 60 | p1 | (T)14 | 14 | 121259 | 121272 |
| SD | 61 | p1 | (T)12 | 12 | 127239 | 127250 |
| SD | 62 | p1 | (T)12 | 12 | 128854 | 128865 |
| SD | 63 | p1 | (A)12 | 12 | 129158 | 129169 |
| SD | 64 | p1 | (A)10 | 10 | 129691 | 129700 |
| SD | 65 | p1 | (T)17 | 17 | 130072 | 130088 |
| SD | 66 | p1 | (T)12 | 12 | 133144 | 133155 |
| SD | 67 | p4 | (GAGG)3 | 12 | 133443 | 133454 |
| SD | 68 | p1 | (A)10 | 10 | 138204 | 138213 |
| SD | 69 | p4 | (ATAG)3 | 12 | 148869 | 148880 |
| SD | 70 | p2 | (AT)5 | 10 | 156226 | 156235 |
